# Comparison of the Effects of Enoxaparin and Heparin on Inflammatory Biomarkers in Patients with ST-segment Elevated Myocardial Infarction: A prospective Open Label Pilot Clinical Trial

**Published:** 2014

**Authors:** Somayyeh Nasiripour, kheirollah Gholami, Sarah Mousavi, Abbas Mohagheghi, Mania Radfar, Mohammad Abdollahi, Zahra Khazaeipour, Mojtaba Mojtahedzadeh

**Affiliations:** a *Clinical Pharmacy Department, Faculty of Pharmacy, Tehran University of Medical Sciences and Health Services, Tehran, Iran. *; b *Clinical Pharmacy Department, Faculty of Pharmacy, Tehran University of Medical Sciences and Health Services and Research Center for Rational Use of Drugs, Tehran, Iran. *; c *Department of Clinical Pharmacy and Pharmacy Practice, Faculty of Pharmacy and Pharmaceutical Sciences, Isfahan University of Medical Sciences, Isfahan, Iran.*; d *Department of Cardiology, Shariati Hospital, Tehran University of Medical Sciences, Tehran 14114, Iran.*; e *Faculty of Pharmacy, and Pharmaceutical Sciences Research Centre, Tehran University of Medical Sciences, Tehran, Iran.*; f ^*f*^*Brain and Spinal Cord Injury Research Center, Imam Khomeini Hospital Complex, Tehran University of Medical Sciences, Tehran, Iran.*

**Keywords:** Heparin, Enoxaparin, Acute coronary syndrome, STEMI, Inflammatory biomarkers

## Abstract

Heparin and enoxaparin possess anti-inflammatory properties. We compared the effects of these drugs on inflammatory biomarkers in patients with ST-segment Elevated Myocardial Infarction (STEMI).

Thirty four patients with STEMI randomly separated in two groups and received standard doses of heparin and enoxaparin. The serum concentration of Serum Amyloid A (SAA), C-Reactive Protein (CRP), Interleukin (IL)-6, ferritin and Myeloperoxidase (MPO) were measured at baseline, 12 ,24 and 48 hours after drug administration.

Serum concentrations of SAA (P: 0.02), CRP (P: 0.02) and ferritin (P: 0.01) significantly reduced in heparin group during measurements compared to baseline, circulating levels of IL-6 (P: 0.002), SAA (P: 0.009), CRP (P: 0.01) were significantly decreased in enoxaparin group. The overall difference in inflammatory biomarkers between heparin and enoxaparin group was not significant.

Both heparin and enoxaparine reduced serum levels of inflammatory biomarkers in patients with STEMI. This effect may provide additional clinical benefit of these drugs in the treatment of STEMI patients.

## Introduction

Inflammation has a key role in the pathogenesis of coronary artery plaque destabilization and rupture leading to Acute Coronary Syndrome (ACS) (1). Leukocyte activation, monocyte and neutrophil infiltration result in local and systemic inflammatory responses (2, 3). High circulating levels of inflammatory markers have been associated with negative prognostic values in patients with ACS (4, 5). Biomarkers such as Serum Amyloid A (SAA)(6, 7), Interleukin(IL)-6(8), fibrinogen (9), Tumor Necrosis Factor alpha(TNF-α)(10), CD 40 ligand(11) and Transforming Growth Factor (TGF- beta)(12) predicts mortality in ACS(both ST-segment elevated Myocardial Infarction( STEMI) and Non- STEMI.

Heparin and enoxaparin are used during STEMI to prevent blood clotting. Besides their anticoagulant effects, growing bodies of evidence are showing that these drugs have anti-inflammatory properties (13-29). The anti-inflammatory effects of heparin and enoxaparin also could enhance the clinical effectiveness of their anti-thrombotic potential (30). The ARMADA (Attribution Randomisee enoxaparine/heparine/dalteparine pour evaluer les Marqueurs d’ Activation cellulaire Dans l’ Angor instable) study evaluated anti-inflammatory effects of heparin and Low-Molecular Weight Heparins (LMWHs) in non-STEMI patients. They reported that inflammatory markers was affected more favorably by enoxaparin than heparin(31). Only small studies evaluated specific anti-inflammatory role of heparin but not enoxaparin in patients with STEMI (32). 

The present study aims to assess the effects of heparin and enoxaparin on inflammatory biomarkers including: SAA, IL-6, C-reactive protein (CRP), myeloperoxidase (MPO) and ferritin in patients with STEMI.

## Experimental

*Methods*

The ethic committee of Tehran University of Medical Sciences (TUMS) approved the study protocol. Informed consent was obtained from patients or their next of kin. Our clinical trial had been registered in Australian and Newzealand Clinical Trial Registry (ANZCTR) with a registration ID in ANZCTR of “ACTRN1261100139965”.

This was a prospective open label pilot study that conducted between august 2010 to September 2011 in Cardiac Care Unit (CCU) of “Shariati “hospital, Tehran, Iran. Eligible patients aged >18 years were considered for inclusion if they exhibited 1) Continuous chest pain upon presentation, refractory to nitrates and lasting ≥ 30 min; 2) ST-segment elevation of ≥ 0.2mv in ≥2 contiguous precordial leads, or ≥ 0.1 mv in ≥2 contiguous limb leads , or new left bundle branch block on admission electrocardiogram 3) Elevated serum levels of cardiac markers.

The main criteria for exclusion were: pregnancy, treatment with heparin or enoxaparin for >24 hours before enrollment, treatment with a glycoprotein IIb/IIIa inhibitors, dipyridamole within the previous 2 weeks, treatment with an oral anticoagulant within previous 5 days, acute inflammatory disease (acute arthritis or acute infection) at the time of randomization, massive hemorrhage or blood transfusion, weight > 120 or < 40 Kg, acute renal failure, sepsis, hemoglobin < 8g/dl, patients receiving high dose of corticosteroids and thrombocytopenia within treatment period.

*Treatment protocol*

Patients selected and randomized (simple randomization) to 2 treatment groups. Both groups received standard treatment for STEMI based on our hospital protocol. First group received weight adjusted enoxaparin (1 mg/Kg subcutaneously at 12 hours interval). Second group received heparin (initial bolus 60 unit/Kg, followed by a continuous infusion at 12 units/Kg/h). The infusion of heparin was dose adjusted according to the activated partial thromboplastin time (a PTT). The target a PTT being 1.5 to 2.5 times control, streptokinase was the thrombolytic agent used (1.5 MU IV over 60 min).

All patients received aspirin orally in a dose of 325 mg upon presentation and it was continued as a daily dose (100-325 mg) indefinitely. Further medical therapy including clopidogrel, beta blockers, nitrates, Ca channel blockers, angiotensin converting enzyme inhibitors and statins was prescribed based on hospital protocol.

Patient’s demographic, medical history and laboratory data were collected on pre-designed questionaries. Patient’s clinical and paraclinical characteristics were recorded at baseline as the following: Blood urea Nitrogen (BUN), Creatinin (Cr), WBC count, Platelet count, hemoglobin, Blood pressure, Heart rate, Blood sugar, Ejection Fraction (EF), Electrolyte (Na^+^, K^+^, Ca^2+^, Mg^2+ ^),Creatin kinase MB, Prothrombin Time(PT), a PTT and international Normalization Ratio(INR). 

*Collection of blood samples and biologic measurements*

Venous blood samples were taken at baseline (T0), 12(T1), 24(T2) and 48(T3) hours after drug administration. Blood samples were spun at 1500*g for 10 minutes and then was stored at -80 ^0^C until the time of analysis.

Hs-CRP concentrations were analyzed by an immunoturbidimetric assay (Pars azmun, Tehran, Iran). Ferritin was measured by a chemiluminescence assay (Diasorin-lialison, Stillwater, MN, USA).

SAA and MPO were analyzed using commercially available enzyme-linked immunosorbent assay kit (USCN Lifescience Inc, Wuhan, China). IL-6 was determined by an enzyme-linked immunosorbent assay kit (Abcam, Cambridge, UK).

*Statistical analysis*

The distribution of quantitative data was assessed for normality by one sample Kolmogorov-Smirnov test. t-test was used, for comparing quantitative variables in two groups. Qualitative variables were compared by Chi square test or Fisher's exact test when appropriate. Repeated measurement analysis was conducted for serial comparisons of quantitative variables and comparisons between groups in different times of treatment. Qualitative variables were recorded by frequency and percent and quantitative variables by Mean ± SD (Standard Deviation). All statistical analysis were conducted using SSPS version 11.5 (SPSS Inc., Chicago, IL, USA) and significance was defined as p-value of <0.05.

## Results

*Baseline characteristic*

During the recruiting period 34 patients with STEMI were enrolled in the study, 17 in each group. There were no statistically significance differences between groups in regard to clinical characteristics and laboratory data. Baseline inflammatory biomarkers were not significantly different in both groups (Tables 1, 2).

**Table 1:** Clinical characteristics of qualitative variables in groups of intervention

	**Enoxaparin**	**Heparin**	**P**
**Gende**	**1female (5.9%)**	**2female (11.8%)**	**1** ♠
	**16men (94.1%)**	**15men (88.2%)**	
CAD History	9 (52.9%)	7 (41.2%)	0.4
Smoking	8 (47.1%)	6 (35.3%)	0.4
Hyperlipidemia	7 (41.2%)	3 (17.6%)	0.1
Hypertension	5 (29.4%)	2 (11.8%)	0.3♠
Diabetes Mellitus	4 (23.5%)	3 (17.6%)	1♠

CAD: Coronary Artery Disease

P: Level of significance (<0.05)

Chi-Square Test

♠ Fisher's Exact Test

**Table 2:** Clinical characteristics of quantitative variables in groups of intervention

	**Enoxaparin** **N=17**	**Heparin** **N=17**	**p** ♦
Age	56.6±13.3	56.5**±**12.5	0.9
BMI	25.3±3.3	24.9±2.5	0.7
WBC	13317.0±3030.9	13141.2±1757.8	0.4
RBC	4.8±0.5	4.8±0.5	0.8
Hb	14.6±1.4	14.3±1.4	0.5
PLT	214823.5±58314.0	223352.9±50131.0	0.6
BUN	19.1±4.5	18.4±4.2	0.6
Cr	1.1±0.1	1.1±0.1	1.0
Systolic BP	130.0±13.3	125.7±14.7	0.3
Diastolic BP	79.1±9.3	75.7±6.8	0.2
Heart Rate	79.3±5.5	77.3±7.6	0.3
Blood Sugar	132.3±33.1	141.8±51.3	0.5
Ejection Fraction	42.0±6.6	39.3±4.7	0.2
CKMB	342.1±203.8	454.0±235.5	0.1
AST	28.9±6.7	29.2±18.8	0.9
Alk ph	229.3±52.6	213.5±49.3	0.3
sodium	140.0±2.5	141.7±3.0	0.08
potassium	4.2±0.3	4.5±0.3	0.6
Calcium	8.9±0.4	9.1±0.6	0.2
Magnesium	2.0±0.3	2.2±0.3	0.058
PT	14.0±2.9	25.6±48.5	0.3
PTT	29.9±6.4	30.2±6.5	0.9
INR	1.1±0.1	1.1±0.2	0.9
SAA T0	50.9±14.5	51.3±14.1	0.9
MPOT0	53.7±20.0	61.3±29.4	0.3
CRP T0	115.7±148.4	99.9±111.3	0.4
IL-6 T0	34.6±28.8	29.2±28.4	0.5
Ferritine T0	213.3±97.8	275.8±136.3	0.09

BMI: Body Mass Index, Hb: hemoglobin, PLT: Platelet, BUN: Blood Urea Nitrogen, Cr: Creatinine, BP: Blood Pressure, CKMB: Ceratin Kinase MB, AST: Aspartat aminotransferase, Alk Ph: Alkalin phosphatase, PT:Prpthrombin Time, PTT:Partial Thromboplastin Time, INR:International Normalization Ratio, SAA: Serum Amyloid A, MPO: Myeloperoxidase, CRP: C-Reactive Protein, IL-:Interleukin , T0:measurement at baseline(before intervention)

P: Level of significance (<0.05)

♦ t-test

Changes of inflammatory biomarkers in four steps of measurements are presented in Table 3.

**Table 3:** Changes of inflammatory biomarkers in 4 steps of measurements in heparin and enoxaparin group.

	**Group**	**T0**	**T1**	**T2**	**T3**	**P (changes in 4 steps)**	**P(comparing 2 groups)**
SAA	Heparin	51.3±14.1	43.1±16.0	40.7±12.4	42.2±12.6	0.02	0.5
	Enoxaparin	50.9±14.5	48.4±13.7	43.6±14.7	42.5±12.9	0.009	
MPO	Heparin	61.3±29.4	56.1±29.1	53.8±35.9	58.6±36.1	0.6	0.5
	Enoxaparin	53.7±20.0	58.5±25.3	46.3±21.1	52.1±16.8	0.5	
CRP	Heparin	99.9±111.3	89.6±89.1	68.4±50.2	35.4±30.4	0.02	0.8
	Enoxaparin	115.7±148.4	82.5±112.5	50.2±80.7	45.9±50.0	0.01	
IL-6	Heparin	29.2±28.4	28.4±23.6	20.9±18.3	17.9±17.2	0.08	0.9
	Enoxaparin	34.6±28.8	27.0±24.9	20.4±24.2	18.6±21.4	0.002	
Ferritine	Heparin	275.8±136.3	263.2±117.9	247.4±107.8	229.1±89.5	0.01	0.1
	Enoxaparin	213.3±97.8	189.1±82.9	187.2±76.4	181.3±72.7	0.2	

T0: baseline, T1: 12 h after intervention, T2:24 h after intervention, T3: 48 h after intervention

SAA: Serum Amyloid A, MPO: Myeloperoxidase, CRP: C-Reactive Protein, IL-:Interleukin

P: Level of significance (<0.05)

**Figure 1:**
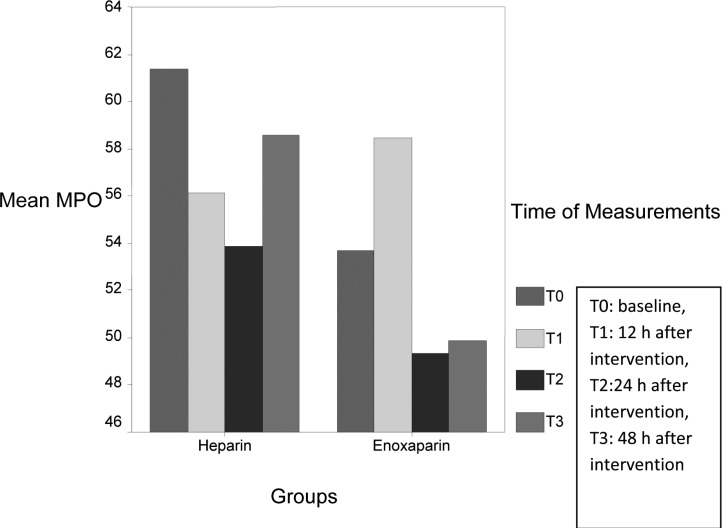
Change of myeloperoxidase(MPO) values in heparin and enoxaparin groups.

Circulating levels of SAA (P: 0.02), CRP (P: 0.02) and ferritin (P: 0.01) significantly reduced in heparin group during measurement, however circulating levels of IL-6 (P: 0.002), SAA (P: 0.009), CRP (P: 0.01) were significantly decreased in enoxaparin group. MPO levels had variable changes during measurement (Figure 1), however the levels were lower in enoxaparin group (P: 0.5). The overall difference in inflammatory biomarkers between heparin and enoxaparin group were not significant.

## Discussion

The current study has shown that both heparin and enoxaparin reduced serum levels of inflammatory biomarkers in patients with STEMI. This effect may provide additional clinical benefit in the treatment of STEMI patients.

Release of Acute Phase Reactant proteins (APR) play a causative role in ACS. Elevated circulating levels of inflammatory biomarkers have prognostic value (4). High levels of hs-CRP increased risk of long term cardiovascular mortality in STEMI patients (33) and American Heart Association (AHA) recommend it as a biomarker for risk stratification in cardiovascular disorder (34). SAA had higher values in STEMI patients and together with hs-CRP had been associated with a major number of complicated lesions in patients with ACS (6, 35). Some of the commonly used drugs can reduce the inflammation process in ACS (36-38). The potential of heparin as an anti-inflammatory drug is supported by several clinical trials in various models of inflammatory disease (13-29). Heparin inhibits the function, expression or synthesis of adhesion molecules, cytokines, angiogenic factors and complements (39, 40). Also some* in-vitro* and *in-vivo* studies show that LMWHs possesses anti-inflammatory effects (18, 20, 22, 41-43). Based on our result, both drugs significantly reduced serum levels of CRP, SAA which regard to the role of these APR proteins worthy to be considered.

The ARMADA study The ARMADA study compared effects of unfractioned heparin (UFH), enoxaparin and dalteparin in 141 patients with unstable angina or non- STEMI. In this study enoxaparin decreased level of von willberand factor (an APR protein) significantly compared to heparin. The authors concluded that enoxaparin had superior and equivalent efficacy over UFH in unstable angina (31). James *et al.*, evaluated the safety and efficacy of treatment with glycoprotein IIb/IIIa inhibition in addition to aspirin, low molecular-weight heparin and its influence on coagulation and inflammation in patients with unstable angina. Baseline levels of creatinine, C-reactive protein (CRP), troponin T (TnT) and N-terminal pro-brain natriuretic peptide (NT-proBNP) were analysed, but non of the drugs or combination of them reduced in flammatory markers in this study (44). Oldegren *et al*., evaluate the prolonged effect of dalteparine in unstable coronary artery disease, patients were received dalteparin 120 IU Kg(-1) s.c. twice daily for 5-7 days and randomized to placebo (n=285) or gender and weight-adjusted doses of dalteparin (5,000 or 7,500 IU) twice daily (n=270) for 3 months. Dalteparin persistently depressed coagulation activity but Interleukin-6, C-reactive protein and fibrinogen levels were unaffected by dalteparin treatment (45). Our study was conducted in STEMI patients and in this setting both drugs was successful in reducing levels of inflammatory biomarkers. Although it is mentioned that pattern of activation of inflammation is different in non-STEMI and STEMI patients (46) and it is possible that heparin and enoxaparin have different mechanism of actions and work in a dissimilar way in ACS.

IL-6 is a well-known cytokine that have important role in inflammatory response (47, 48). High levels of IL-6 also increase the risk of mortality in STEMI patients (8), however both drugs reduced levels of IL-6 but the reduction was significant in enoxaparin group. Why enoxaparin is more effective than heparin on IL-6 is question to be answered in future studies.

Myeloperoxidase is a hemoprotein that abundant in rupture plaques and useful as a clinical tool in coronary artery disease (CAD) (49), however it is inferior to CRP as a marker for risk stratification. The levels of MPO had variable changes during our measurements, recent study shows for the first time the existence of diurnal variations in MPO levels in STEMI patients (higher at night), so time of blood sampling is important and it could be the reason of fluctuation of MPO levels in our study.

## Conclusion

Heparin and enoxaparin are one of the important parts of ACS treatment, based on the role of inflammatory process in STEMI patients and potential of these drugs to suppress inflammatory biomarkers. It is probable that part of the benefit of these drugs in STEMI patients is because of their anti-inflammatory properties. Due to the small sample size conducted a similar study with a larger sample size is recommended. 
